# Sustainable bioplastics manufacturing from renewable sources

**DOI:** 10.1002/2211-5463.70174

**Published:** 2026-03-18

**Authors:** C. Valeria L. Giosafatto, Marika Avitabile, Michela Famiglietti, Talayeh Kordjazi, Marzieh Moosavi‐Nasab, Odile F. Restaino, Loredana Mariniello

**Affiliations:** ^1^ Department of Chemical Sciences University of Naples Federico II Italy; ^2^ Institute of Food Sciences, National Research Council Avellino Italy; ^3^ Department of Food Science and Technology, School of Agriculture Shiraz University Iran

**Keywords:** bioplastics, film  biological properties, industrial applications, renewable sources, technological attitudes

## Abstract

Fossil‐based material manufacturing has long been linked to the acceleration of climate change through carbon dioxide emissions. In addition to their negative impact on the environment, the depletion of nonrenewable fossil fuels has led to a global demand for sustainable and environmentally friendly alternatives. This has sparked a surge of academic interest in the past few decades on the manufacture of bio‐based materials as substitutes for fossil‐based materials. As sustainability becomes a global imperative, bioplastics are rapidly emerging as a viable alternative to conventional petroleum‐derived plastics. These materials might be manufactured by using polymers from different bio‐based sources such as plants, animal tissues, or can have a microbial origin. Bioplastics not only offer biodegradability, thereby reducing long‐term environmental impact, but also possess various functional properties that make them suitable for diverse applications, including packaging, agriculture, textiles and pharmaceuticals. This review focuses on new developments in bioplastics regarding their material, processing, and applications. Recent developments in the preparation of bioplastics are reported, highlighting the distinct properties of each type of material according to the polymers of origin. Special attention is given to the film‐forming properties, the barrier functionality, thermal stability, and compatibility with the bioactive compounds, supported by recent empirical findings.

## Graphical abstract

Bioplastics are manufactured by using polymers from different bio‐based sources. These novel materials not only offer biodegradability but also possess various functional properties that make them suitable for diverse applications. Recent developments in the preparation of bioplastics are reported, highlighting the distinct properties of each type of material according to the polymers of origin.

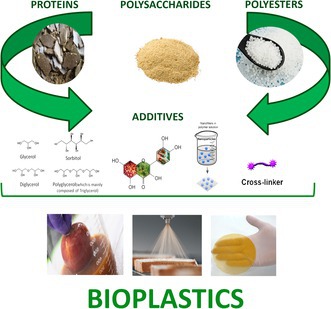

**Abbreviations**
CNC: cellulose nanocrystals
CNF: cellulose nanofibers
DPPH: 2,2‐diphenyl‐1‐picrylhydrazyl
EO: essential oil
FFS: film‐forming solutions
FRAP: ferric reducing antioxidant power
PBAT: poly(butylene adipate‐co‐terephthalate)
PBB: protein‐based bioplastics
PBS: poly(butylene succinate)
PCL: poly‐ε‐caprolactone
PE: polyethene
PEG: poly(ethylene glycol)
PET: poly(ethylene terephthalate)
PHA: polyhydroxyalkanoates
PHBV: poly(3‐hydroxybutyrate‐co‐3‐hydroxyvalerate)
PLA: poly(lactic acid)
SCL: secalin
SOC: seed oil cake
TEAC: trolox equivalent antioxidant capacity
WPI: whey protein isolate

## Plastics vs bioplastics

Plastic pollution has emerged as one of the most pressing environmental challenges of the 21st century, permeating ecosystems from the deepest oceans to the highest mountains. The widespread use of plastics in nearly every sector of modern life, from packaging and consumer goods to agriculture and health care, has led to an unprecedented release of these synthetic polymers into natural systems. Characterized by the accumulation of plastic debris in the environment, this global crisis stems from the remarkable durability and slow degradation rates of the plastic items, also coupled with escalating rates of plastic production and inadequate waste management systems [1]. Addressing plastic pollution requires a multifaceted approach that might involve the actions of single individuals, citizens, businesses, and specific acts of the governments. So far, several approaches have been put into practice worldwide to reduce the impact of plastics on the environment as described below:

- *Reduction of single‐use plastics*: This is the most direct way to tackle the problem. Citizens might reduce the use of unnecessary single‐use plastics (straws, bags, and takeout utensils) and opt for reusable alternatives (water bottles, coffee cups, and grocery bags) [2].
- *Improvement of waste management*: Investing in and implementing robust waste process systems for collection, sorting, and recycling. The increase of waste management infrastructure is a global, crucial issue [3].
- *Promotion of recycling*: Proper recycling practices are essential. Consumers should be aware of what type of plastics are recycled and how they work in their local waste management systems [4].
- *Legislation and policy*: Advocate for policies that reduce plastic production, encourage reuse, and improve waste management [5].
- *Cleanup efforts*: Participating, as citizens, in beach or river cleanup campaigns to remove the existing plastic pollution from the environment [6].
- *Shift societal values*: Fostering a ‘plastic‐free’ culture that emphasizes refusal, reduction, reuse, and repair is important for long‐term change [7].
- *Develop alternative materials*: Research and development program to support new biodegradable and compostable alternatives or bioplastic items that might replace conventional plastics. Bioplastics are often discussed as a promising alternative to conventional plastics in the ongoing battle against plastic pollution. However, their role as plastic alternative is complex and their use does not represent a simple one‐to‐one solution. To understand their potential and limitations, it is essential to make a differentiation between the different types of bioplastics and to evaluate their impact on the environment as well as their lifecycle [8, 9, 10, 11, 12].

These alternative materials can be produced by means of renewable resources, whose exploitation is imperative as a cost‐effective approach for large‐scale production of biopolymers [13]. Renewable resources include agro‐industrial residues such as food waste, dairy waste, and grain waste, and used as feedstocks for commercial biopolymer production [14]. The solid waste is pretreated (physical, chemical, and/or biological) for conversion of its complex organic molecule components (e.g., lignin, cellulose, and starch) into sugars, which are later subjected to microbial fermentation for biopolymer production [15].

## How can bioplastics act as an alternative to plastic pollution?

The mounting environmental and health challenges posed by synthetic plastic pollution have triggered a global push toward sustainable alternatives. Bioplastics offer a potential solution to plastic pollution by providing a more sustainable alternative to traditional plastics, which are typically derived from fossil fuels. Bioplastics can be made from polymers derived from renewable biomass sources, such as plants. They are all bio‐based but just some of them are wisely designed to be completely biodegradable or compostable, to also reduce the reliance on landfills and minimize the long‐term environmental impact. The use of bio‐based bioplastics has the primary benefit of shifting away from finite fossil resources. This can lead to a lower carbon footprint during production. Besides, as plants absorb CO_2_ during their growth, the use of plant‐based biopolymers to produce bioplastics potentially might drive the achievement of carbon neutrality, or even negative emissions in some scenarios, depending on the feedstock and production process (European Bioplastics, 2025 https://www.european‐bioplastics.org), thus aligning with the global efforts to mitigate climate change. In addition, biodegradable bioplastics promise to break down in specific environments, thereby reducing the accumulation of persistent plastic waste. This is particularly appealing for single‐use applications where collection and recycling are challenging [16, 17]. It is worth pointing out that many common biodegradable bioplastics are designed to degrade effectively in industrial composting facilities where controlled conditions (high temperatures, specific moisture, and microbial activity) accelerate the process. Under these optimal conditions, they can break down within a few months. This can divert waste from landfills and create valuable compost. Moreover, if a bioplastic fully biodegrades, it theoretically reduces the long‐term risk of microplastic formation, a major concern connected with the use of conventional plastics.

However, the term ‘bioplastic’ is an umbrella term encompassing a diverse range of materials. They are generally categorized in two main ways:

1. *Bio‐based plastics*: These plastics are wholly or partially derived from renewable biomass sources, such as corn starch, sugarcane, cellulose, and vegetable oils. Crucially, *not all bio‐based plastics are biodegradable*. Bio‐based polyethene (Bio‐PE) and bio‐based poly(ethylene terephthalate) (Bio‐PET), for example, are chemically identical to their fossil‐fuel counterparts and behave similarly in the environment, meaning they are *not* inherently biodegradable and will persist if littered or landfilled [18].
2. *Biodegradable plastics*: These plastics are designed to decompose into natural substances (such as water, carbon dioxide, and biomass) through the action of microorganisms [19]. Not all biodegradable plastics are bio‐based; some are derived from fossil fuels but are engineered to be biodegradable. Some bioplastics are both bio‐based and biodegradable (e.g., starch‐based material, such as the one commercialized by Novamont, Italy, under the tradename Mater‐Bi^®^), while others are only one or the other. For example, despite being biodegradable, poly‐ε‐caprolactone (PCL) and poly(butylene adipate‐co‐terephthalate) (PBAT) are made from fossil fuels.

## Biomolecules useful for bioplastics manufacturing

**Fig. 1:**
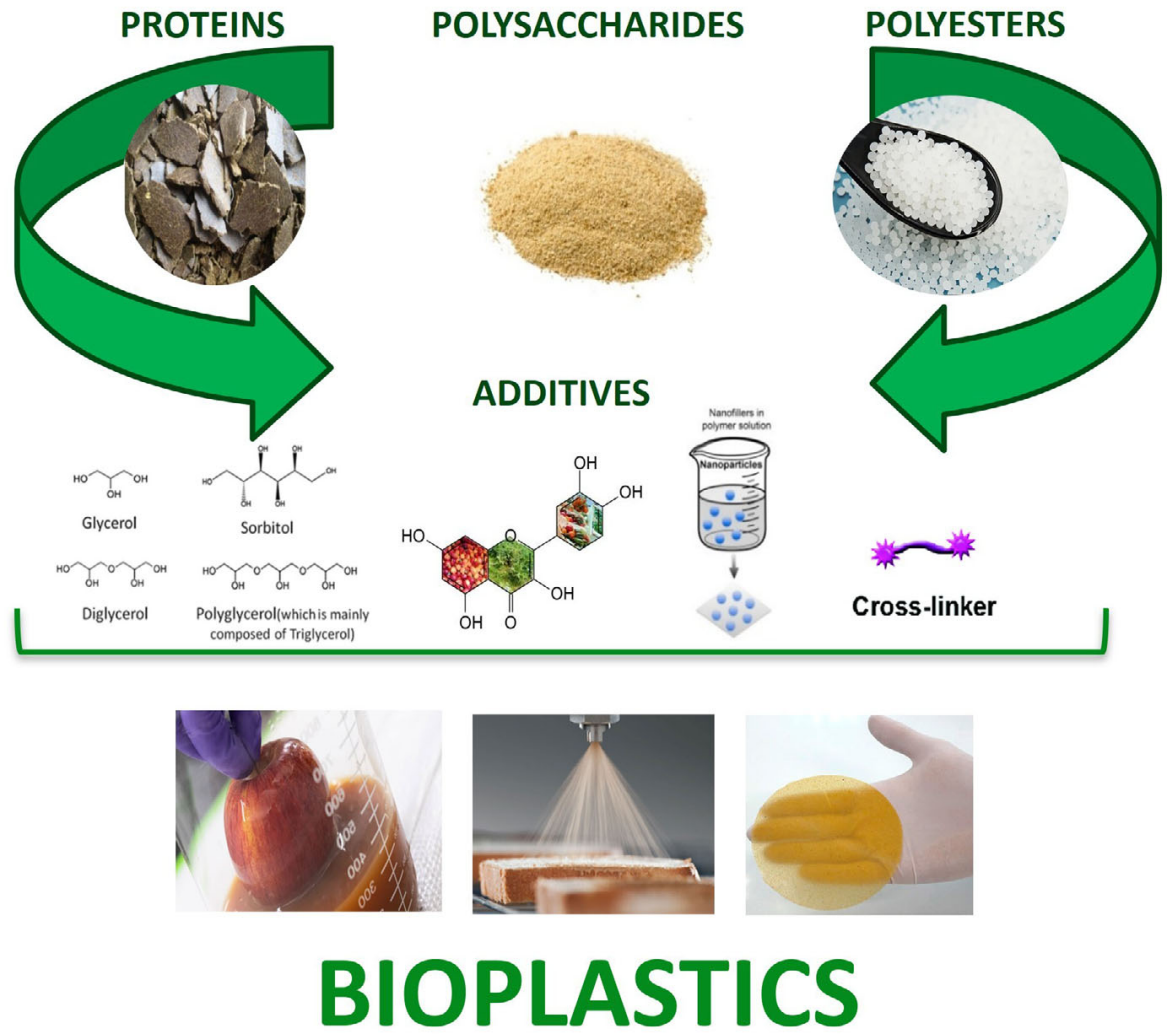
Biomolecules and additives used to produce bioplastics.

Bioplastics can be prepared from different sources and polymers. Mostly, they are manufactured from polysaccharides, proteins, and polyesters (Fig. 1). In order to strengthen the matrix of the materials and to improve their biological and technological attitude, several additives can be added to the film‐forming solutions (FFSs) (Fig. 1). In the following paragraphs, an overview of the different types of molecules used for bioplastics manufacturing is reported.

### Protein‐based bioplastics (PBB) and applications

Bioplastics, particularly those derived from proteins, offer eco‐friendly packaging options with potential applications in food preservation, biomedical devices, and agricultural films. Protein‐based bioplastics are valued for their film‐forming capabilities, biodegradability, and compatibility with functional bioactive compounds such as probiotics, prebiotics, and polyphenols [20, 21, 22]. Among the proteins, plant‐derived ones are widely studied due to their accessibility, low cost, and functional versatility. The most prominent sources include soy protein isolate, wheat gluten protein, corn zein, pea protein, and proteins from seed oil cakes (SOCs). Soy protein isolate offers excellent film‐forming ability, good oxygen barrier properties, and biodegradability. However, the low hydrophilicity of these bioplastics limits water resistance, which is commonly addressed by adding crosslinkers, plasticizers, or nanofillers [20]. On the other hand, wheat gluten proteins are rich in gliadins and glutenins. They form films with high tensile strength and oxygen barrier properties. The poor water resistance and brittleness of such protein‐based bioplastics can be improved by blending with egg white protein, tartaric acid, or polysaccharides [22]. The zein is a hydrophobic prolamin capable of forming cohesive, glossy films. It is particularly useful for incorporating lipophilic compounds. However, the mechanical fragility of the corn zein‐based materials often necessitates reinforcement through blending [20, 22]. Legume proteins are very much exploited for the bioplastic sector; as a matter of fact, proteins from pea [20] as well as grass pea, rich in legumin and vicilin, were used as a matrix for bio‐based materials considering that they have good emulsification and antioxidant properties [23]. Nonetheless, these legume protein bioplastics suffer from rigidity and brittleness, which are usually improved using plasticizers such as glycerol or starch composites [24]. Regarding SOCs, nearly 600 million tons of oilseeds, mostly to produce edible oil, were produced worldwide in 2018–2019 (USDA, 2018b). However, the by‐products produced after the oil extraction, called seed oil cake (SOC), often make up around 50% of the original seed weight [25, 26, 27]. Quite recently, it has been demonstrated that the proteins extracted from SOCs possess good film‐forming properties. Argan, sunflower cardoon, and hemp SOCs were also exploited to manufacture films potentially useful in different fields—from the food sector to the agriculture ones to prepare mulching sheets [25, 26]. Utilizing these agricultural by‐products is an innovative way to recycle them into valuable new materials, contributing to a biorefinery approach [26, 27]. Recent research has highlighted the potential of valorizing agri‐food biowastes and by‐products as alternative protein sources for bioplastic production. For instance, Álvarez‐Castillo *et al*. [28] demonstrated that proteins recovered from agri‐food industrial by‐products can be successfully processed into films with promising barrier and mechanical properties, thus offering a sustainable route for packaging applications [28]. Similarly, Álvarez‐Castillo *et al*. [29] emphasized how biowaste‐derived proteins could be transformed into functional and biodegradable plastics, aligning with circular economy principles and waste valorization strategies [29]. It is worthy to say that proteins from argan SOCs were able to produce films that for their satisfying physicochemical and biological properties (hydrophobicity, wound healing, and antioxidant) could be applied also as face masks in the satisfying cosmeceutical field [27]. There is also a study that reports SOCs to obtain novel biomaterials [30]. It is important to point out that the biodegradability features and ability to incorporate bioactive compounds make the materials produced from SOCs attractive for niche applications [20]. As far as animal‐derived proteins are concerned, it is possible to say that they often possess superior mechanical properties and barrier functionality compared to plant proteins. The most exploited animal proteins that are worth mentioning are Whey Protein Isolate (WPI): WPI offers excellent oxygen and oil barrier properties. Its mild processing conditions make it ideal for bioplastics that incorporate sensitive bioactive molecules. However, it remains hydrophilic and requires plasticizers to reduce the brittleness of the derived films [22, 30, 31]. Rossi Marquez *et al*. [32] prepared edible films using glycerol as a plasticizer to overcome the brittleness of WPI‐based coating for fresh‐cut fruits and vegetables, observing a reduction in both hardness and chewiness of all the uncoated samples after 10 days of storage. However, Corrado *et al*. [33] were able to produce nano‐biocomposite material based on the use of poly‐3‐hydroxybutyrate‐co‐hydroxyhexanoate nanoparticles within a scaffold of WPI‐based films. In addition, Abdalrazeq *et al*. [34] successfully produced bioactive packaging materials made of WPI denatured under alkaline conditions that were further functionalized with essential oil (EO) extracted from Thymbra (*Satureja capitata, L*.) [34], one of the most popular Palestinian wild plants, and with pecan extract nut shells [35] that were able to exert antimicrobial activity against *Enterococcus faecalis* and the food pathogen bacterial strain *Salmonella enterica* subsp. *enterica* ser. *Typhimurium* envisaging their possible use for the production of edible systems to extend food shelf‐life. Sodium caseinate derived from casein forms transparent and flexible films. Their barrier properties can be enhanced with natural antimicrobials or zein coatings. Crosslinking with agents such as genipin further improves the performance of the derived bioplastics (Table 1) [20]. Also, gelatin and collagen, extracted from connective tissues, form highly transparent and elastic films. While gelatin exhibits good gas and aroma barrier properties, its thermal instability and water solubility limit certain applications [36]. However, fish gelatin extracted from fish collagen has higher solubility and a lower gelling point than mammalian gelatin, making it suitable for cold‐set applications [36, 37, 38]. Talking about different sources, egg white proteins, possessing globular proteins such as ovalbumin and ovotransferrin can form heat‐resistant, antioxidant‐rich films. UV or enzymatic crosslinking enhances their bioplastic barrier and mechanical properties [20].

**Table 1:** Main chemical and enzymatic crosslinking agents used to strengthen the matrix of bioplastics.

Crosslinking agents	Mechanism of action	References
Chemical agents		
Formaldeyde	Reaction of the aldeyde group with amino acids present in proteins to form a methylol adduct, then converted to a Schiff base	[39]
Glyoxal and glutaraldehyde	Reaction of the dialdeyde group with hydroxyl or amino acid groups present in polysaccharides or proteins to form acetal or hemiaminal linkages	[39]
Genipin	Reaction with hydroxyl or amino acid groups present in polysaccharides or proteins	[40]
Citric acid	Esterification reactions with hydroxyl groups of polysaccharides	[41, 42]
Enzymatic agents		
Microbial transglutaminase (EC 2.3.2.13)	Catalysis of an acyl transfer between glutamine and lysine residues present in proteins to form ε‐(γ‐glutamyl) lysine isopeptide bonds	[23, 27, 34, 43, 44, 45, 46, 47]
Papain (EC 3.4.22.2)	Catalysis of an acyl transfer from a thiol ester (e.g., N‐acetyl‐dl‐homocysteine thiolactone, AHTL) presents in proteins to form peptide‐like bonds.	[45, 47]
Laccase (EC 1.10.3.2)	Oxidization of a variety of phenolic substrates, performing one‐electron oxidations, leading to crosslinking.	[48]

Quite recently microbial proteins—produced by algae, bacteria, fungi, and yeast—are gaining attention as sustainable, rapidly renewable protein sources for bioplastic preparation. Among them, it is possible to list (i) Algal Proteins (e.g., spirulina): Algae provide abundant, fast‐growing protein sources that can be transformed into bioplastics with good antioxidant and UV‐barrier properties [21]: (ii) Fungal Mycoprotein: mycelium from fungi can be processed into flexible films with antimicrobial attributes. These bioplastics are biodegradable and can be used in food wrapping or biodegradable tableware [21]; (iii) Bacterial Proteins: recombinant and wild‐type bacteria such as *Bacillus subtilis* and *Corynebacterium glutamicum* produce high‐protein biomass that can be processed into functional packaging materials. However, scalability remains a challenge due to the necessity of the development of large‐scale biotechnological production processes [21].

### Polysaccharide‐based bioplastics and applications

Polysaccharide‐based films have gained significant attention as viable alternatives to conventional petroleum‐based plastics due to their biodegradability, renewability, and functional versatility. These materials, derived from natural sources, offer promising solutions for sustainable packaging, food preservation, and biomedical applications. Among the various polysaccharides investigated, cellulose, starch, alginate, pectin, and chitosan have exhibited excellent film‐forming properties, making them suitable for diverse industrial applications. Polysaccharide‐based films possess unique characteristics that make them attractive also for environmentally friendly applications [49], such as their natural abundance and complete biodegradability that ensure minimal environmental impact, addressing concerns over plastic pollution. Moreover, polysaccharide‐based films can be engineered to have barrier properties against oxygen and moisture, enhancing their potential use in food packaging. While native polysaccharides often require structural modifications or the incorporation of additives to improve mechanical strength and flexibility, research has focused on optimizing their functional attributes to expand their range of applications. Plasticizers are commonly used to improve flexibility and reduce brittleness; fillers and reinforcing particles enhance strength, thermal stability, and barrier properties; and blending with other polymers can help balance toughness and processability [50].

#### Chitosan‐based films

Among polysaccharides, chitosan has emerged as a particularly promising biopolymer due to its remarkable biodegradability, antimicrobial properties, and excellent film‐forming capability. Chitosan derives from chitin, which is abundantly found in marine crustaceans such as shrimp and crab shells. Its linear polysaccharide structure, composed of glucosamine and/or N‐acetylglucosamine units, provides intrinsic biological functions that enhance its utility in packaging and biomedical fields [51]. Chitosan‐based films are valued for their natural antimicrobial activity, which helps in extending the shelf‐life of perishable products by inhibiting bacterial growth. Additionally, these films exhibit excellent oxygen barrier properties, preventing oxidative deterioration of foods. Due to their inherent biocompatibility and edibility, chitosan films have been widely explored as coatings for fresh products, as protective layers for pharmaceuticals, and for biodegradable packaging solutions [52]. Their monosaccharide composition allows for effective hydrogen bonds between the chitosan chains, which contributes to film integrity, high tensile strength, and barrier efficiency. The presence of amine groups (‐NH_2_) gives chitosan intrinsic antimicrobial properties, making it highly effective in controlling spoilage microorganisms and food‐borne pathogens. Chitosan‐based films have found widespread applications in the food industry, where they serve as biodegradable packaging materials and edible coatings. These films not only help extend the shelf‐life of perishable food items but also offer moisture retention, texture preservation, and antimicrobial protection. Edible chitosan coatings are particularly beneficial for fruits, vegetables, seafood, and dairy products, acting as a barrier against microbial contamination and oxidative degradation [53]. Given its biocompatibility, nontoxic nature, and wound‐healing properties, chitosan is extensively used also in medical and pharmaceutical applications. Chitosan films are utilized in wound dressings, drug delivery systems, tissue engineering scaffolds, and surgical implants. Their ability to prevent bacterial infections, promote cell adhesion, and control drug release has made them an essential component in advanced therapeutic materials. Moreover, chitosan‐based nanocomposite films have gained traction in antimicrobial wound dressings, offering sustained release of bioactive compounds such as silver nanoparticles, antibiotics, or growth factors [54]. Chitosan‐based biodegradable films are also being explored as eco‐friendly alternatives in agriculture, primarily in mulching applications [55]. These films serve as soil protection layers, preventing water evaporation and improving crop yield while minimizing reliance on synthetic plastic‐based mulch. Additionally, chitosan‐based films are being investigated for their ability to act as plant immune stimulators, enhancing resistance against fungal and bacterial infections [56]. This function has led to their development as bioactive coatings for seeds and plant protection films, reducing the need for chemical pesticides. Chitosan films are increasingly being incorporated into cosmetic formulations, particularly in the development of biodegradable face masks, skin‐care patches, and transdermal delivery systems. Their ability to retain moisture, improve skin hydration, and offer antimicrobial protection makes them a viable option for natural and sustainable beauty products [57]. Despite all these properties, modifications of chitosan‐based films are often required to improve mechanical strength and moisture resistance. The incorporation of plasticizers, crosslinking agents, and nanostructures has been widely investigated to enhance the durability and functional properties of chitosan‐based bioplastics. In the food industry, chitosan‐based edible films are particularly advantageous for packaging fresh foods while maintaining the safety and quality of the products [58]. The ongoing advancements in chitosan‐based film preparation indicate a growing interest in developing tailored formulations for specific industrial applications, bridging the gap between eco‐friendly material design and large‐scale commercial feasibility. Further research in nanocomposite approaches, hybrid biomaterials, and biodegradable blends continues to expand the possibilities of chitosan as a sustainable alternative to conventional plastics. To further enhance their functionality, essential oils, antioxidants, or nanomaterials are often incorporated into chitosan films. While chitosan‐based films hold significant promise across multiple industries, challenges related to mechanical strength, water resistance, and large‐scale commercial production remain. Future research will be focused on enhancing the functionality of chitosan films through chemical modifications, hybrid formulations, and nanotechnology integration. Emerging studies suggest that combining chitosan with other biodegradable polymers (such as polylactic acid, starch, or protein‐based materials) may provide improved flexibility, durability, and cost‐effectiveness, paving the way for industrial‐scale deployment of sustainable chitosan‐based bioplastics [26].

#### Pectin‐based films

Pectin‐based films have gained considerable attention as biodegradable and renewable alternatives to petroleum‐derived plastics. Pectin, a natural polysaccharide primarily extracted from citrus peels and apple pomace, is widely used in food and pharmaceutical industries due to its gel‐forming ability, biocompatibility, and nontoxic nature [59]. Its film‐forming capability makes it an excellent candidate for eco‐friendly packaging solutions, particularly in food preservation and biomedical applications. Pectin‐based films exhibit remarkable oxygen barrier properties, which help reduce oxidative degradation of packaged food products. In fact, pectin‐based films are extensively used in food packaging, particularly as edible coatings for fruits, vegetables, and dairy products. Their biodegradability and nontoxic nature make them ideal for sustainable food preservation, reducing reliance on synthetic plastic packaging Additionally, active pectin films incorporating antioxidants, essential oils, or antimicrobial agents have been developed to enhance food safety and extend shelf‐life [9]. Due to their biocompatibility and bioadhesive properties, pectin‐based films are widely explored in drug delivery systems, wound dressings, and tissue engineering scaffolds. Their ability to control drug release and promote cell adhesion makes them valuable in advanced therapeutic applications. Recent studies have investigated pectin‐based nanocomposites for antimicrobial wound dressings, offering sustained release of bioactive compounds [60]. Pectin‐based biodegradable films are being studied as eco‐friendly alternatives in agriculture. These films help retain soil moisture, improve crop yield, and reduce synthetic plastic waste. Additionally, pectin‐based coatings for seeds have been developed to enhance germination rates and protect against microbial infections, reducing the need for chemical treatments [61]. Pectin‐based films are increasingly incorporated into cosmetic formulations, particularly in biodegradable face masks, skin‐care patches, and transdermal delivery systems. Their moisture‐retaining and skin‐hydrating properties make them suitable for natural and sustainable beauty products [62]. The issue of the high water solubility of chitosan‐based films might be modified through crosslinking techniques or blending with other biopolymers such as chitosan, alginate, or starch, improving their mechanical strength and moisture resistance. The incorporation of plasticizers such as glycerol or sorbitol further enhances their flexibility and durability, making them suitable for preparing bioplastics with various industrial applications [62]. While pectin‐based films offer significant advantages, challenges related to mechanical strength, water resistance, and large‐scale commercial production still remain. Thus, future research might be focused on enhancing their functionality through chemical modifications, hybrid formulations, and nanotechnology integration. Combining pectin with other biodegradable polymers (such as chitosan, starch, or cellulose derivatives) might be a solution to improve flexibility, durability, and cost‐effectiveness, thus paving the way for industrial‐scale deployment of sustainable pectin‐based bioplastics [63]. The study of Giosafatto et al. [64] was the first to report the use of pectin extracted from *Foeniculum vulgare* for the development of biopolymer films incorporating phaseolin protein. This pioneering work laid the foundation for exploring unconventional plant matrices in the creation of biodegradable materials with promising mechanical and functional properties [65, 66].

#### Starch‐based films

Starch‐based films are among the most widely studied biodegradable materials due to their renewability, low cost, and excellent film‐forming ability. Starch is a natural polysaccharide composed of amylose and amylopectin, typically extracted from sources such as corn, potato, rice, and cassava. Its molecular structure allows for the formation of transparent, flexible films with good oxygen barrier properties, making it a strong candidate for sustainable food packaging [67]. Starch‐based films are also being explored as active packaging systems, incorporating antimicrobial agents, antioxidants, or essential oils to enhance food safety and shelf‐life. Beyond food packaging, starch‐based films are being investigated for pharmaceutical, agricultural, and biomedical uses, including drug delivery systems, seed coatings, and wound dressings. Their biodegradability and nontoxicity make them environmentally friendly alternatives to synthetic polymers, aligning with global efforts to reduce plastic waste. Starch‐based films have demonstrated remarkable versatility across multiple sectors due to their biodegradability, film‐forming ability, and compatibility with active compounds. Their applications span from food packaging to biomedical and agricultural uses, making them a cornerstone in the development of sustainable materials [68]. Starch‐based films are widely used in the food industry as edible and biodegradable packaging materials. Their excellent oxygen barrier properties help reduce oxidation and spoilage, especially in dry food products. However, their high‐water sensitivity limits their use with moist foods. To address this, starch films are often blended with lipids, essential oils, or nanomaterials to enhance water resistance and antimicrobial activity. These films can also serve as carriers for antioxidants and preservatives, extending shelf‐life and improving food safety [69, 70].

In the pharmaceutical field, starch‐based films are employed in drug delivery systems, wound dressings, and tissue engineering scaffolds. Their biocompatibility and ability to encapsulate bioactive compounds make them ideal for controlled drug release. Starch nanoparticles and nanocomposites have been developed to improve mechanical strength and modulate degradation rates, enabling targeted therapeutic applications [71]. Additionally, starch‐based hydrogels and films are being explored for transdermal patches and oral dissolvable films. Starch‐based films are gaining traction in agriculture as biodegradable mulch films and seed coatings. These films help retain soil moisture, regulate temperature, and suppress weed growth, thereby enhancing crop yield. Unlike conventional plastic mulches, starch‐based alternatives decompose naturally, reducing environmental impact [72]. Moreover, starch films can be infused with fertilizers or pesticides for controlled release, minimizing chemical runoff and improving nutrient efficiency. In the cosmetics sector, starch‐based films are used in biodegradable facial masks, skin patches, and delivery systems for active ingredients. Their moisture‐retaining and skin‐friendly properties make them suitable for hydrating treatments and antiaging formulations. These films can be engineered to release botanical extracts, vitamins, or peptides gradually, offering a sustainable alternative to synthetic polymers in personal care products [73]. However, native starch films are inherently brittle and highly sensitive to moisture, which limits their mechanical performance and water resistance. To overcome these limitations, researchers have developed modified starch‐based films through the incorporation of plasticizers (e.g., glycerol and sorbitol), crosslinking agents, and reinforcing fillers such as cellulose nanofibers or agricultural residues. These modifications significantly improve the tensile strength, elasticity, and water vapor barrier properties of the films, enabling their use in a broader range of applications [74, 75].

### Polyester‐based bioplastics and applications

Polyester‐based bioplastics are a major class of biopolymers synthesized through polycondensation or ring‐opening polymerization of hydroxy acids or their derivatives and characterized by the presence of ester functional groups within their backbone structure [73, 75]. Their properties and biodegradability are significantly influenced by the chemical structure of the monomers used; in fact, their structure offers the potential for their hydrolytic and/or enzymatic degradation that is highly dependent on environmental conditions [73, 75]. As a matter of fact, these materials can be either bio‐based, biodegradable, or both, depending on their monomer origin and polymerization processes used to prepare them. Polyester‐based bioplastics are generally categorized into natural polyesters and synthetic biodegradable polyesters. In fact, depending on the types of monomers incorporated, polyester‐based bioplastics can be classified as aliphatic, aromatic, or copolymers. The inherent chemical structure of many polyester‐based bioplastics, especially those with heteroatom linkages in the main chain, supports selective chemical recycling strategies, allowing recovery of monomers, oligomers, or other valuable intermediates. Polyhydroxyalkanoates (PHAs) are natural polyesters produced intracellularly by various microorganisms as energy and carbon storage macromolecules [73]. PHAs are fully biodegradable and can be tailored to exhibit a wide range of mechanical and thermal properties depending on their monomeric composition. Also, a variety of biodegradable polyesters have been developed through chemical synthesis, including poly(lactic acid) (PLA), poly(butylene succinate) (PBS), PBAT, and PCL. These materials have been engineered to achieve specific property profiles suitable for applications in packaging, agriculture, biomedical devices, and more. Polyesters exhibit considerable structural diversity and synthetic flexibility, which has led to the creation of materials with tailored properties for various industrial applications. Advances in catalytic and enzymatic technologies are expected to drive the large‐scale commercialization of such recycling processes [76, 77].

#### Aliphatic polyesters

Aliphatic polyesters are derived from aliphatic monomers, which include linear or branched alkanes that might also contain carboxyl and hydroxyl functional groups. Notable examples include Poly(lactic acid) (PLA), PCL, and PHAs. These polymers are generally characterized by high biodegradability and are extensively used in packaging, agriculture, and biomedical applications. However, their mechanical strength and thermal stability are often lower than those of the aromatic polyesters.

#### Aromatic polyesters

Aromatic polyesters incorporate aromatic monomers such as terephthalic acid, which introduce rigidity into the polymer backbone. These materials typically exhibit improved thermal and mechanical properties, making them suitable for more demanding applications. However, the presence of aromatic rings often reduces biodegradability. Poly(ethylene terephthalate) (PET) is not naturally biodegradable but is a durable plastic that resists natural degradation, making it non‐biodegradable under typical environmental conditions [76, 77].

#### Aliphatic–aromatic–copolyesters

To balance biodegradability with mechanical performance, aliphatic–aromatic–copolymers are produced by copolymerization of aliphatic and aromatic monomers. These hybrid materials, such as PBAT, combine the flexibility and biodegradability of aliphatic units with the strength and durability of aromatic segments. This category has gained attention in the development of flexible packaging films and compostable plastics [77].

#### Production process and sources of polyester‐based bioplastics

Polyester‐based bioplastics, including PLA, PHAs, and PCL, are primarily derived from renewable biological sources such as microorganisms or agricultural waste.

PHAs are intracellular polyesters synthesized by bacteria under specific nutrient conditions, particularly when the carbon source is in excess. Different kinds of bacteria, including *Ralstonia eutropha*, *Bacillus sp*., and *Pseudomonas spp*., have been identified as PHA producers. Once produced, PHAs are extracted from microbial biomass through methods such as solvent extraction, digestion, flotation, and supercritical fluid extraction. PHAs are categorized based on the monomer chain length into short‐chain (C3–C5), medium‐chain (C6–C14), and long‐chain PHAs (>C14), each of which shows different mechanical and physical properties.

PLA is a thermoplastic polyester synthesized from lactic acid, which is typically produced through the fermentation of carbohydrate‐rich biomass such as corn starch, sugarcane, or agricultural residues. PLA production involves three main steps: (i) fermentation of sugars to produce lactic acid; (ii) formation of lactide via lactic acid dimerization; and (iii) polymerization of lactide by ring‐opening polymerization.

PCL is produced by ring‐opening polymerization of ε‐caprolactone, which can be derived from saccharides through fermentation followed by oxidation processes. PCL is known for its biodegradability, compatibility with other polymers, and applications in biomedical fields and packaging. Despite being fossil‐based, polyesters such as PBS and PBAT also exhibit significant biodegradability and are often included in the broader class of bioplastics.

Polyester‐based bioplastics, such as PLA, PHAs, and PCL, offer a balanced combination of mechanical strength, thermal stability, and biocompatibility. These materials generally exhibit good tensile strength, though their flexibility and impact resistance may require enhancement through blending or enzymatic modification [78]. In terms of barrier performance, they provide good oxygen impermeability, while water vapor permeability varies depending on the polymer hydrophobicity. Their thermal stability supports common processing methods such as extrusion and casting; however, PLA and PHAs can degrade under elevated temperatures [79]. To improve functionality, these polyesters are often blended with other biopolymers such as starch, gelatin, chitosan, proteins, and lipids, forming composite materials tailored to specific applications. Crosslinking agents such as microbial transglutaminase are also employed to enhance film strength while maintaining safety. Plasticizers such as glycerol and sorbitol improve flexibility and processing by lowering the glass transition temperature, though their concentrations must be optimized to preserve mechanical and barrier properties. Due to their renewable origin, biocompatibility, and versatility, polyester‐based bioplastics are used in diverse sectors including packaging, biomedical devices, agriculture, and consumer goods. In biomedicine, PCL and PHAs are applied in drug delivery systems, surgical implants, and tissue engineering scaffolds owing to their controlled degradability and biological compatibility. PLA is widely used in disposable packaging and biodegradable films that require controlled conditions in industrial composting facilities [80]. Additionally, polyester‐based bioplastics are critical for developing edible and active packaging systems. PLA and PHAs can be cast into biodegradable coatings for products like fruits, cheeses, and confections, enhancing microbial protection and appearance. By incorporating natural antioxidants, antimicrobials, or flavor agents, these films can function as active packaging, extending shelf‐life. Smart packaging systems based on polyester matrices are also under development. These respond to environmental cues, such as pH, humidity, or temperature, by releasing functional compounds, thereby improving food safety and quality [79]. Despite their potential, several limitations decrease the widespread application of PLA and PHA. PLA, for example, exhibits brittleness and low impact resistance, restricting its use in applications requiring flexibility and mechanical durability. Moreover, the production of these bioplastics remains economically challenging, largely due to high feedstock and processing costs. In response to these limitations, ongoing research is focused on enhancing the functional performance and sustainability of polyester‐based bioplastics. The incorporation of nanomaterials and bio‐based fillers into polyester matrices has shown promise in improving mechanical strength, thermal stability, and barrier properties. The valorization of marine biomass and agricultural residues as alternative feedstocks presents a viable route to reduce production costs and environmental burden. Furthermore, advances in metabolic engineering and synthetic biology are enabling the development of genetically optimized microbial strains for more efficient biosynthesis of polyesters [81, 82].

### Additives to produce bioplastics

#### Plasticizers

Plasticizers are essential for giving bioplastics the flexibility and processability needed for various applications. They function by weakening the forces between the polymer chains, thus increasing the material plasticity [83]. This is especially important in hydrocolloid‐based bioplastics, where the hydrophilic nature of the hydrocolloids can lead to stiffness and weak mechanical properties [84]. Selecting the appropriate plasticizers is critical for tailoring the final bioplastic mechanical, thermal, and degradation characteristics [85]. These plasticizers interact with biopolymeric materials, causing structural modifications to the three‐dimensional matrix [86]. Incorporating plasticizers typically enhances the flexibility of protein films by loosening the protein network. However, this also tends to increase gas permeability. Plasticizers can also enhance processability, and this could be useful as biopolymers have low degradation temperatures and might break down during processing because the energy required to split the polymer chains is close to the energy for degradation, Plasticizers insert themselves between the polymer chains of the bioplastic materials thus altering the interactions, enhancing the lubrication and increasing free volume [87]. Plasticizers seem also to affect processability and biodegradation, although research on this aspect is still ongoing [88].

The addition of plasticizers changes different bioplastic properties. For example, water vapor permeability (WVP) of bioplastics generally increases as the concentration of plasticizers rises. This is because plasticizers weaken internal hydrogen bonding and expand the intermolecular spacing within the film structure [89]. Water is considered a natural plasticizer because it can form hydrogen bonds with polar functional groups of biopolymers, such as hydroxyl and amine groups. Some other hydrophilic compounds such as polyols, carbohydrates, and amines may also interact with polar groups of the biopolymer chains, producing a plasticizing effect. Examples include glycerol, sorbitol, saccharose, trimethylene glycol, and polyethylene glycol, which have a polar nature, are compatible with the biopolymer matrix, while their small size allows them to penetrate the macromolecular network [90]. Glycerol easily inserts itself between polymer chains, increasing free volume and chain mobility. The addition of plasticizers like glycerol enhances mechanical properties such as tensile strength and elongation at break, necessary, for example, for starch‐based films. Films containing glycerol absorbed more water and at a faster rate than those with sorbitol. Mechanically, glycerol provided more effective plasticization, enhancing the flexibility of the films by increasing the mobility of amylose and amylopectin chains, despite potential recrystallization effects [91]. Increasing glycerol concentration in alginate/pectin composite films resulted in lower TS, higher water solubility, increased moisture content, and better elongation at break [92]. Other polyols such as poly(ethylene glycol) (PEG) and fructose also serve as plasticizing agents [92]. The impact of various polyols, glycerol, ethylene glycol, polyethylene glycol, and propylene glycol, on the mechanical and surface properties of chitosan films has been assessed with attention to plasticizer volatility, as lower volatility enhances film stability during application and storage. Glycerol and polyethylene glycol demonstrated superior plasticization efficiency and long‐term stability among the tested plasticizers compared to ethylene glycol and polyethylene glycol. A concentration of 20% (w/w) of either glycerol or polyethylene glycol was sufficient to produce flexible chitosan films with stable properties maintained for up to five months [93]. Plasticizer concentration and hydrophilicity significantly influence the moisture affinity of cassava starch films too [94].

Polyols were also used in protein‐based films: Glycerol served as a plasticizer in fish protein films, resulting in decreased opacity, color intensity, and glass transition temperature [95]. Conversely, films that were plasticized with ethylene glycol, sucrose, or sorbitol were too brittle and fragile for practical applications. PEG mainly affected TS, whereas glycerol had a more significant impact on elongation at break. The results underscored glycerol's effective plasticizing role, which is due to its high hydrophilicity that disrupts internal hydrogen bonding within the protein matrix, decreasing cohesive forces and enhancing molecular mobility [96]. In addition, glycerol does not bioaccumulate or persist in the environment, reducing its potential for long‐term harm. In fact, Benitez *et al*. [97]. demonstrated that this molecule improved the biodegradation of bio‐based films in seawater, which facilitates waste management. This can be linked to glycerol's high water solubility and its ability to serve as a carbon source for certain seawater‐dwelling microbes [97].

Sorbitol, a sugar alcohol with a larger molecular weight (~182 g·mol^−1^), is another effective plasticizer. Compared with glycerol, sorbitol‐plasticized starch films show higher TS at equivalent loadings, for example, at 15 wt% sorbitol, TS reached 28.35 MPa versus 9.59 MPa with glycerol; however, it produces lower elongation, indicating a more modest plasticizing effect [98]. In chitosan–gelatin blends, glycerol at equal ratios produced films with higher TS (4.04 MPa) and moderate elongation (~26%) compared to sorbitol (3.94 MPa tensile strength, ~68% elongation at break in a 1:2 blend) [99].

Finally, polyamines such as spermidine and spermine were found to act as plasticizers in protein matrix films. For example, in the presence of the polyamines spermidine and spermine, zeta potential and Z‐average characterization of native and heat‐denatured bitter vetch protein particles in film‐forming solutions showed that spermidine can specifically function as a cationic plasticizer to produce protein‐based films. On the other hand, it was discovered that spermine merely reduced the minimum amount of glycerol required to function as a plasticizer by forming hydrogen bonds with proteins [100].

#### Nanofillers

The incorporation of fillers into bioplastics represents a pivotal strategy for minimizing their inherent limitations, such as poor mechanical strength, high gas permeability, and low thermal stability, thereby broadening their applicability in diverse sectors [101]. Nanofillers, characterized by their nanoscale dimensions (typically less than 100 nm), offer a substantial surface‐to‐volume ratio, and consequently, a large matrix/filler interfacial area, which changes the molecular mobility, the relaxation behavior, and consequently, leads to improved thermal, mechanical, and barrier properties of the resulting biocomposites [102, 103]. The production of these nanofillers includes a wide range of techniques tailored to obtain specific materials with the desired characteristics. The selection of an appropriate method of nanofiller production is crucial for achieving optimal performance in the final bioplastic products. To date, the ‘top‐down’ and the ‘bottom‐up’ approaches are two strategies used in nanotechnology: in the first one nanometric structures are obtained by size reduction in bulk material, including techniques such as photolithography, nano molding, and nanofluidics; in the second one, they are fabricated in a controlled manner by thermodynamic mechanisms from individual atoms or molecules capable of self‐assembling, balancing attraction and repulsion forces between them to form more functional supramolecular structures [104].

The functionalities of nanofillers vary based on their composition, size, surface area, concentration, shape, and affinity with the matrix. They can be divided into two primary groups according to their chemical composition: inorganic and organic fillers. The first group includes calcium carbonate (Ca_2_CO_3_), kaolin (hydrous aluminosilicate), silicon dioxide (SiO_2_), titanium dioxide (TiO_2_), and montmorillonite; while the second group consists of carbon‐based nanofillers and natural nanofillers derived from chitosan, chitin, lignin, and cellulose [105].

A further classification of nanofillers relates to their three‐dimensional geometry. Based on their 3D structure, they are divided into four groups: spherical nanostructures are considered 0D structures, also known as nano‐cluster materials or nano‐dispersion; 1D structures are nanoscale composites measuring at one dimension, typically tube‐shaped with lengths of 100–1000 nm and thicknesses of a few nanometers. The composites of 2D structures are nanoscale in two dimensions and are also referred to as nano‐sheets, while 3D nanostructures exist at the nanoscale in three dimensions and can form polycrystalline systems [106].

The addition of cellulose nanocrystals (CNC) and cellulose nanofibers (CNF) in amylose‐based films, for example, exerts a distinct effect on bioplastics: CNF significantly improves tensile strength and Young's modulus; conversely, the presence of CNC reduces tensile strength while enhancing elongation at break due to increased polymer mobility [107].

Chitosan nanoparticles obtained by the gelation method and incorporated into a fish gelatin matrix to produce bio‐nanocomposite films significantly improved the performance of the films in terms of mechanical and barrier characteristics: a remarkable increase in the TS and elastic modulus, and a significant decrease in the water vapor permeability [108]. Mesoporous silica nanoparticles have even been reported to improve the tensile strength and the elongation at break of enzymatically crosslinked bitter vetch protein‐based films, as well as enhance gas and water vapor barrier properties [109]. Carbon nanotubes and carbon nanofibers were used with successful results to improve the conductivity, thermal, mechanical, and gas barrier properties of thermoplastic biopolyesters such as poly(3‐hydroxybutyrate‐co‐3‐hydroxyvalerate) (PHBV) and PCL [110].

In addition to reinforcing nanofillers, various types of nanostructures have been designed to provide active or smart properties to the system, including antimicrobial and antifungal activities, enzyme immobilization, biosensing, oxygen scavenging, and drug delivery. Some can even serve multiple overlapping applications [111]. The use of nanomaterials in food packaging has garnered considerable attention due to the potential for creating films with antimicrobial properties that can help control the growth of pathogens, thereby enhancing the shelf‐life of food products [112]. Nanostructures based on silver, titanium dioxide, chitosan, and carbon nanotubes (CNTs) have been studied for their properties as growth inhibitors and killing agents against a wide range of microorganisms responsible for food spoilage. Titanium dioxide (TiO2) serves as a photocatalytic disinfecting material for surface coatings, as it promotes the peroxidation of the polyunsaturated phospholipids in microbial cell membranes [113]. Meanwhile, chitosan has been proposed for its ability to interact with the negative charges on the membrane cells of microorganisms, which alters their permeability and eventually leads to cell lysis [114]. The addition of iron‐based nanoparticles (Fe_3_O_4_) has increased the antimicrobial capacity of chitosan‐pectin films against pathogenic Gram‐negative and Gram‐positive bacteria, in addition to functioning as a potential sensor for food packaging due to their electric and magnetic property [115]. Indeed, nanocomposites have even been explored as biosensors for smart food packaging to detect microorganisms, toxic proteins, heavy metals, and degraded products in food and beverages. With their capability to detect the presence of pathogens, chemical compounds, and toxins in food products, nanocomposites offer a promising and efficient way to monitor food spoilage, providing real‐time information on freshness [116, 117].

#### Bioactive compounds

While bioplastics are advantageous because they come from renewable resources and, in some cases, are biodegradable, their functionality can be improved by adding bioactive compounds. These compounds can provide beneficial properties such as antimicrobial, antioxidant, and anti‐inflammatory effects, broadening the uses of bioplastics in areas such as food packaging, medicine, and agriculture. Incorporating bioactive agents into bioplastic matrices is a promising strategy for developing materials with better functionality and sustainability [118].

Nature contains a wide variety of active components from different parts of plants, animals, and soils. Recently, interest in consuming bioactive compounds has increased because of the several health benefits they offer for disease prevention [119]. Many studies have focused on extracting and characterizing these bioactive compounds from various sources and using them as natural ingredients in food products. Likewise, the food packaging industry is shifting from the traditional approach of making coatings and films ‘as inert as possible’ to developing active and smart packaging that can extend food shelf‐life, improve safety, and maintain high product quality [120].

Packaging effectively shields food products from external contamination. It prevents their chemical, physical, and biological changes during preparation and storage. Using active and functional packaging is one of the strategies to extend shelf‐life by reducing gas exchange, respiration, and oxidative reactions of food products [121].

Different matrices can be used to incorporate compounds as antimicrobial or antioxidant agents, including proteins, lipids, polysaccharides, or composites.

Among the most studied bioactive compounds there are phytochemicals, which are secondary metabolites of plants, such as alkaloids and phenolic compounds, known for their antimicrobial and antioxidant activities [122]; antimicrobial agents produced by microorganisms, including antimicrobial peptides such as bacteriocins, valued for their ability to withstand high temperatures and acidic environments; and natural components found in animals, like lysozyme, also known as muramidase or N‐acetylmuranidase, recognized for its effectiveness against several pathogenic bacteria [119].

Curcumin, a polyphenolic compound with known antioxidant and antimicrobial activities, has been successfully integrated into gelatin‐based films, suitable for food packaging applications, demonstrating notable efficacy against pathogenic microorganisms such as *E. coli* and *L. monocytogenes*, and showing strong antioxidant activity comparable to ascorbic acid [123]. Similarly, essential oils like those extracted from oregano and thyme have been incorporated into chitosan‐alginate‐based films, where they inhibit microbial growth, improving the shelf‐life of food products [124].

The method of incorporating these bioactive agents plays a crucial role in determining their effectiveness. Direct blending into the film‐forming solution remains the most straightforward approach, though it may lead to volatility and degradation of thermolabile compounds. Advanced strategies, such as nanoencapsulation and the use of emulsified systems (e.g., nanoemulsions or Pickering emulsions), offer improved stability and enable controlled release of active molecules. These systems can enhance bioavailability while minimizing undesirable interactions with the polymer matrix [125, 126].

Using plant extracts instead of isolated single compounds can be more advantageous in certain applications due to their lower production cost and the synergistic effects among the various bioactive constituents. These synergistic interactions often enhance the overall functional performance, such as antioxidant or antimicrobial activity, beyond what is achievable with individual purified molecules, making extracts a cost‐effective and functionally potent option for bioactive film development.

Chitosan‐based bioplastic films were successfully developed using anthocyanin‐rich extracts from *Callistemon citrinus* flowers [8]. Rich in polyphenols, the extract enhanced antioxidant activity and reduced film hydrophilicity, suggesting the films' potential for sustainable packaging with added antioxidant functionality. Similarly, CH‐based films containing a dried olive leaf extract were obtained, showing, besides antioxidant properties, an antimicrobial effect against three bacterial strains involved in food spoilage [127].

On the other hand, Mirpoor *et al*. [128] produced and evaluated the mechanical, antioxidant, and antibacterial characteristics of PHA‐based bioplastics functionalized with phloretin, a dihydrochalcone, member of the flavonoid class and present in a variety of fruits and vegetables especially apples [129]. Functionalization of PHA‐based polymers led to a reduction in the growth of food‐borne pathogens (*Listeria monocytogenes* ATCC 13932) and the acquisition of antioxidant activity (in a dose‐dependent manner) as determined by 2,2‐diphenyl‐1‐picrylhydrazyl (DPPH), Trolox equivalent antioxidant capacity (TEAC) Ferric Reducing Antioxidant Power (FRAP). Ultimately, apple samples were packed in the functionalized PHA films for 24, 48, and 72 h, and the stabilization of the apple samples was observed to be significantly affected. The findings make it possible to use phloretin as a functionalizing agent in the formulation of bioplastics, particularly for food packaging [128]. Eventually it is worth pointing out the use of egagropili fibers to strengthen the matrix of bioplastics [25, 130, 131, 132]. Egagropili which are fibrous balls from *Posidonia oceanica* are regarded as wastes because they proliferate along sandy beaches, provoking bad odors and, therefore, the municipalities are forced to remove them at negligible costs. Quite recently, egagropili lignin/carbohydrate complex has been suggested as a powerful antioxidant additive to strengthen and enhance the gas barrier qualities of biodegradable materials, which may be helpful for packaging oxidation‐sensitive perishable goods [130, 131, 132].

#### Chemicals and enzymes as crosslinking agents

Crosslinking is essential to enhancing the water resistance and mechanical strength of bioplastics [133, 134]. Chemical crosslinkers as well as enzymatic agents such as transglutaminase have been used to improve structural integrity (Table 1) [133, 134]. In the bioplastic sector, aldehyde‐based crosslinkers, such as formaldehyde, glyoxal, and glutaraldehyde, are greatly used even though they are highly toxic and limit the application scope of protein products (Table 1) [39]. In fact, concerns have been raised about the potential safety risks associated with conventional chemical crosslinking agents [41]. By acting as a chemical crosslinker, citric acid can be considered a safe molecule to enhance the mechanical, thermal, and water resistance of bio‐based plastics, especially those made of starch. Through a process known as esterification, citric acid reacts with starch molecules to produce crosslinks that improve starch's overall functionality. Citric acid was successfully utilized to reduce the number of hydroxyl groups in the film structure made of maize starch polymer molecules by crosslinking them [41]. This led to an improvement in film water resistance [41]. Sagnelli *et al*. [42] have also demonstrated that the crosslinked amylose‐only starch coming from transgenic lines of barley possessed composting properties identical to those of the control, suggesting that the modified starch functions similarly to regular starch. The information demonstrates that using engineered starch as a raw material to produce all‐natural bioplastic is feasible. This example highlights that not all chemical crosslinkers compromise biodegradability, and that careful selection of the crosslinking agent can preserve environmental compatibility [42]. On the other hand, using genipin, a natural nontoxic chemical crosslinking agent, results in more deformable pea protein systems, produced by the thermomoulding fabrication process, with a lesser capacity to absorb water, when compared to uncrosslinked bioplastics. It is worth pointing out that genipin reacts with primary amines, and in this sense, pea proteins, presenting a high content of arginines and lysines, can be effectively crosslinked by this molecule [133] (Table 1).

**Fig. 2:**
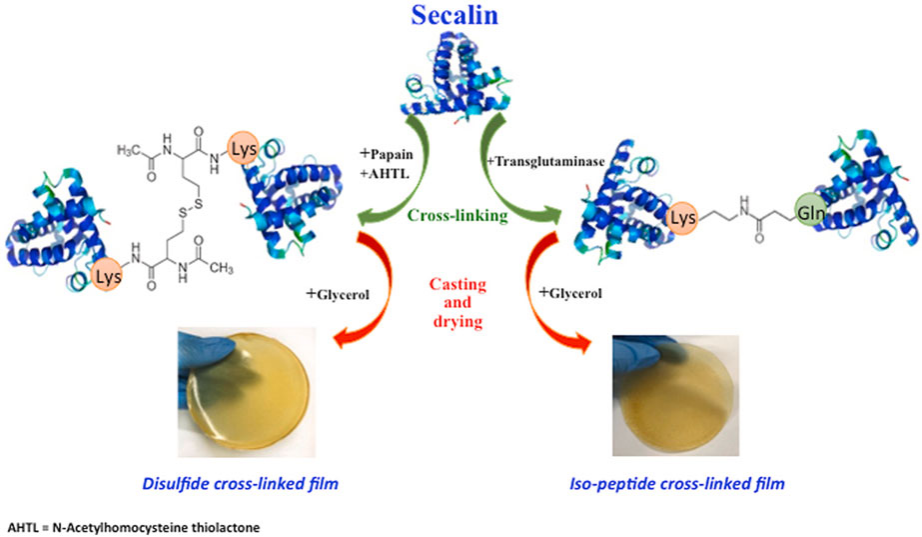
Microbial transglutaminase and papain enzymes used as crosslinking agents in protein‐based bioplastics [45]. AHTL, N‐acetyl‐dl‐homocysteine thiolactone. Fig. reproduced from Ref. [45].

As far as the use of enzymes, enzymatic crosslinking is considered a safer and more desirable alternative, as enzymes enable specific reactions under gentle conditions and yield nontoxic products (Table 1). Protein crosslinks can be formed either directly through covalent bonds between reactive side chains of amino acids or indirectly, with the help of a small added molecule that bridges polypeptide chains. In this regard, it is worthy to highlight the enzyme transglutaminase (TG; EC 2.3.2.13), which forms ε‐(γ‐glutamyl)lysine isopeptide bonds by catalyzing an acyl transfer between glutamine and lysine residues; glutamine serves as the acyl donor and lysine as the acyl acceptor [40]. In particular, the microbial isoform (mTG) has been extensively reported to produce edible materials/bioplastics based on a protein matrix [43, 44, 45, 135, 136, 137]. Quite recently, it has been shown that heat‐denatured whey protein‐based films may benefit from mTG‐mediated modification. The degree of mTG‐mediated crosslinking has been shown to affect the films' mechanical and barrier qualities by increasing the elongation at break and decreasing the Young's modulus. The decrease in tensile strength indicates that the mTG also makes the materials more rigid. Furthermore, following the mTG‐catalyzed crosslinking, the barrier properties against CO_2_ and O_2_ were enhanced. It is also important to emphasize that the enzymatic modification had no effect on the film gastric digestion, indicating the potential application of such materials in various industrial domains, including the food and pharmaceutical industries [12]. mTG was also able to improve the mechanical, hydrophilicity, gas barrier, and thermal features of films made of cardoon proteins that were shown to act as both acyl‐acceptor and acyl‐donor of mTG [138]. Very interestingly, peanuts were found to be preserved by the novel materials as seen by the reduction of the peroxidase value and water content when they were wrapped in sachets made from crosslinked cardoon biopolymers [138]. Papain (EC 3.4.22.2), a plant‐based sulfhydryl protease, can also mediate indirect protein crosslinking. Beyond its known proteolytic role across pH 4 to 7, papain can in alkaline conditions facilitate the transfer of an acyl group from a thiol ester (e.g., N‐acetyl‐dl‐homocysteine thiolactone, AHTL) to an amino compound, forming peptide‐like bonds (Fig. 2). These newly attached thiol esters may then engage in spontaneous disulfide bonding with another polypeptide, effectively acting as crosslinkers. Disulfide bonds are well‐known for stabilizing protein structures, enhancing resilience to extreme environmental conditions. A notable example is keratin, a hydrophobic protein found in hair and feathers, rich in cysteine residues and disulfide linkages. Thus, modifying biopolymer structures by introducing disulfide bonds through papain‐assisted incorporation of thiol groups could be a promising strategy for improving the characteristics of edible film matrices.

Qazanfarzadeh *et al*. [45] demonstrated that the rye protein from secalin was able to be modified by means of both mTG and papain (Fig. 2). Secalin is an alcohol‐soluble protein fraction extracted from rye grains. SCL contains four major polypeptides (high molecular weight > 100 kDa, ≈7%; γ‐75 kDa, ≈46%; ω‐50 kDa, ≈17%; γ‐40 kDa, ≈24%) rich in glutamine and proline [46] and, only recently has it been studied in relation to its functionality in food systems and shown to give rise to films potentially edible [45, 47]. While the resulting films had superior mechanical, barrier, and water resistance qualities, crosslinked SCL demonstrated better emulsifying and foaming qualities. SCL‐based films' elongation at break (200%), Young's modulus (200 MPa), surface hydrophobicity, and barrier qualities (water permeability halved, oxygen permeability tenth, and carbon dioxide permeability twentieth) were all improved by AHTL/papain‐mediated crosslinking. In contrast, films made with mTG crosslinked SCL exhibited a higher tensile strength (TS) (4 MPa) and a lower swelling index (83%). These findings confirm that enzymatic crosslinking can be tailored to achieve specific mechanical or functional outcomes, although trade‐offs between strength, flexibility, and degradability must be carefully considered [66]. In addition, the papain‐modified secalin was exploited to prepare bio‐pesticides [46] to replace the synthetic agro‐chemicals that are very pollutant for the environment. In fact, the FFSs prepared from secalin were able to deliver Bordeaux mixture on the leaves of *Rosa chiniensis* Jacq. opening new horizons in the manufacture of environmentally friendly pesticides. Therefore, a novel green technique for controlling plant diseases could be a leaf coating made of a protein‐based FFS with little pesticide content. Also, laccases are widely used for bioplastics production. Laccases (EC, 1.10.3.2) are found in plants, fungi, insects, and bacteria with varying roles [48, 139]. In plants, the known roles of laccases include lignification, wound healing, and polymerization of seed coat. They can create sophisticated lignin‐based materials, which is comparable to their function in the natural synthesis of lignin [48] (Table 1). Lignin is one of the bio‐based compounds that garners a lot of interest due to its unique characteristics, polyaromatic structure, and abundance in plant biomass. Laccases are promising green instruments that can broaden the commercial inclusion of lignin‐based products and improve the use of lignin in industrial processes [48].

### Bioplastics degradation and waste management

One of the main goals of the actual bioplastic research and manufacturing processes is to develop materials that will be less persistent in the environment than the petrol‐based polymers as well as easier to be biodegradable and less toxic. The possibility of a bioplastic to degrade and the rate of degradation depend on the physicochemical properties of the material, like the polymer molecular weight, the thickness, and the crystal structure, as well as on the conditions of disposal [140]. From a waste management point of view, the bioplastics actually present on the market could be classified in two classes: bio‐based or biodegradable. Generally, bioplastics obtained from renewable biomasses, like PE or PET, are considered bio‐based but they might have low biodegradability in the environment, as they are constituted by units that chemically resemble the fossil‐based polymers [112]. Biodegradable bioplastics like PLA and PHAs might, instead, be biodegraded in the environments (abiotic processes), in soil or in aquatic systems, through the hydrolytic action of the water. They can also dissolve by simple exposure to air or sunlight through abiotic fragmentation that led to the formation of particles that are not toxic for the environment [140]. The degradation process of these materials might involve also biotic mechanisms. Initially, abiotic factors break down the polymer chains into oligomers and monomers; subsequently, microbial activity facilitates further degradation, converting these smaller molecules into carbon dioxide, water, methane (under anaerobic conditions), and biomass. Nevertheless, the process of biodegradation depends on the type of bioplastics and the degradation conditions. For example, PLA exhibits limited biodegradation in ambient soil and aquatic environments due to its high crystallinity and hydrophobic nature. However, under industrial composting conditions, where temperatures are maintained around 58 °C with high humidity, PLA can achieve significant degradation within a few months. In contrast, PHAs demonstrate more rapid biodegradation across diverse environments, including soil and marine settings, attributed to their more amorphous structure and susceptibility to microbial enzymes. Starch‐based and PLA bioplastics might be degraded thanks to the activity of microorganisms (biotic processes), at specific conditions of pH, temperature and humidity. Both procaryotic and eucaryotic strains of the species of *Pseudomonas*, *Erythrobacter*, *Enterobacter*, *Streptomyces*, *Aspergillus*, or *Bacillus* have been found to synthesize enzymes able to degrade bioplastics like PBS and PLA, as they are able to produce enzymes like N‐acetyl‐glucosaminidase, esterase, acid phosphatase and phosphohydrolase [141, 142]. The biodegradation occurring through microbial actions generally leads to complete mineralization producing water, carbon dioxide, ammonium ions, hydrogen or nitrogen [140]. But the biodegradation requires studies on the type and characteristics of the bioplastic, contemporary with studies on the possible microorganisms or communities to be employed in the process. In bioplastic waste management, some traditional methods of disposal such as recycling or incineration are still nowadays used together with more eco‐friendly systems based on microorganism action, like composting or landfilling. As the bioplastic degradation is slow, in any case, their disposal at the end of their use, is preferred through composting methods rather than in landfills or simply in soils. Composting is safe, economic and might turn the polymers into humus to fertilize the soil thanks to the microbial action, while landfilling procedures of bioplastic disposal have the drawbacks of producing greenhouse gases and necessitate occupying large areas of soil [142]. Besides composting procedures performed at controlled, high temperatures push the microbial enzymatic degradation of bioplastic, accelerating the rate.

### Biodegradable plastic global market

According to the United Nation Environment Program every year from 19 to 23 million tons of plastic wastes are dumped worldwide into water environments. This causes widespread plastic pollution that influences ecosystems and natural habitats, causing environmental, social, economic, and health risks for all living species up to human beings [143]. Together with a strong necessity of designing new, more efficient and secure ways of plastic item disposal and waste management, there is nowadays a global scientific and industrial interest in replacing plastic with biodegradable materials. This has driven the global market to invest in this sector up to USD 5.92 billion in 2024, with an estimated Compound Annual Growth (CAGR) of 16.40%, with a projection up to USD 18.44 billion by 2031 [144]. The European Union is nowadays the biggest market for biodegradable plastic items with a percentage of 42.60% in 2023, thanks to a high ecological awareness of the citizens and to several directives and bans approved by the European Commission on single‐use and on oxo‐biodegradable plastic products [145]. The North America bioplastic market counts for 29.60% of the total global market. That is because, for example, in the United States, several states or single cities have banned the selling of single‐use plastic items [146], but not a federal law has been approved against petrol‐based plastics. In Asia, the government of Japan, instead, has proposed in 2019 a road map to increase the use of bioplastic items by 2030 [146]. The countries that have approved laws or roadmaps for the substitute the plastic with biodegradable bioplastic are also the ones where the main manufacturing companies are like BASF (Germany), Novamont (Italy), Danimer Scientific, (Georgia, USA), NatureWorks LLC (Nebraska, USA), or Mitsubishi Chemical Corporation (Japan). Starch‐based plastics are the most produced, but PLA, PHA, PBAT, or PBS are widely sold on the market [144]. In the perspective of a market regulation of bioplastic products, standards and certification must be required, as well as quality assurance tests to avoid frauds. For all these reasons, since 2021, the European Community has planned to invest money in policies for the management of bioplastic labeling and certification of the standards [145]. This effort will probably increase also customer awareness and further push for a higher market request for biodegradable plastic products. Besides, the corrected classification and labeling of bioplastics will help also to design new and efficient methods of waste management.

## Conclusions

Bioplastics derived from renewable sources represent a sustainable and functional solution to plastic pollution. Their diversity—spanning plant, animal, marine, and microbial sources—offers rich possibilities for material customization and functional enhancement such as water resistance, biodegradability, and antimicrobial activity. Continuing advancements in encapsulation techniques, material blending, and process optimization have notably enhanced the mechanical and barrier properties of bioplastics, positioning them as key contributors to circular bioeconomy strategies. They not only reduce environmental pollution but also encourage resource efficiency by valorizing waste and supporting closed‐loop systems. These attributes are especially beneficial in sectors like food packaging, agriculture, and biomedical applications, where sustainability and performance are critical [147]. In conclusion, there are numerous advantages to using bioplastics for a variety of purposes, such as promoting agricultural activities, utilizing renewable resources, and lessening the impact on the environment. However, compared to synthetic plastics, the current technological costs of creating bioplastics remain much greater. Since the bio‐based plastics industry is still in its infancy, bioplastics require government backing or action to shield them from synthetic material competition. Furthermore, fragmented supply chains and market competition from synthetic materials complicate expansion. To accelerate the industry's growth and improve competitiveness, governmental policies and financial incentives are essential. Public investment, biodegradability standards, and supportive legislation can catalyze innovation and help bioplastics gain traction. With the right backing, bioplastics could become an integral part of sustainable development and global efforts to combat plastic pollution.

## Conflicts of interest

The authors declare no conflicts of interest.

## Author contributions

CVLG and LM were involved in conceptualization, visualization, and supervision. CVLG, MA, MF, MMN, TK, and OFR were involved in writing – original draft preparation. CVLG, MA, and OFR were involved in writing – review and editing.
